# Exploring the Coordination of Cardiac Ion Channels With Action Potential Clamp Technique

**DOI:** 10.3389/fphys.2022.864002

**Published:** 2022-03-16

**Authors:** Balázs Horváth, Norbert Szentandrássy, Csaba Dienes, Zsigmond M. Kovács, Péter P. Nánási, Ye Chen-Izu, Leighton T. Izu, Tamas Banyasz

**Affiliations:** ^1^Department of Physiology, University of Debrecen, Debrecen, Hungary; ^2^Department of Basic Medical Sciences, Faculty of Dentistry, University of Debrecen, Debrecen, Hungary; ^3^Department of Pharmacology, University of California, Davis, Davis, CA, United States

**Keywords:** cardiac electrophysiology, voltage clamp, action potential voltage clamp, ion current, pharmacology

## Abstract

The patch clamp technique underwent continual advancement and developed numerous variants in cardiac electrophysiology since its introduction in the late 1970s. In the beginning, the capability of the technique was limited to recording one single current from one cell stimulated with a rectangular command pulse. Since that time, the technique has been extended to record multiple currents under various command pulses including action potential. The current review summarizes the development of the patch clamp technique in cardiac electrophysiology with special focus on the potential applications in integrative physiology.

## Introduction

Cardiac action potential (AP) is shaped by numerous ionic currents flowing in and out of the cell through the sarcolemma. The ultimate goal in cellular cardiac electrophysiology is to characterize the dynamics of individual ionic currents and understand how the interplay of these currents will draw the profile of the AP in different experimental conditions. There are two conceptually different methods to determine the profile of a given ionic current during AP. To accomplish this goal in the traditional way, we have to determine the magnitude of each discrete current from moment to moment during the AP. This step is followed by the reconstruction of the AP from the interplay of the individual actors. The traditional way employs rectangular command pulses mapping the channel kinetic parameters and the voltage dependence of the parameters. Based on this information, the profile of the current can be reconstructed during the AP with the help of computer simulation. The other approach, termed the AP clamp method, uses prerecorded AP command and measures the current profile directly during the AP in question. Current experimental methods allow the researcher to record multiple currents under the same AP. The goal of this article is to review the evolution of the voltage clamp method in a classical way employing rectangular command pulse to the sequential the AP voltage clamp technique. We aimed to compare the benefits and disadvantages of the methodical variations of these techniques with special emphasis on the integrative approach.

## The Conceptual Difference Between Traditional Voltage Clamp and Action Potential Voltage Clamp

**FIGURE 1:**
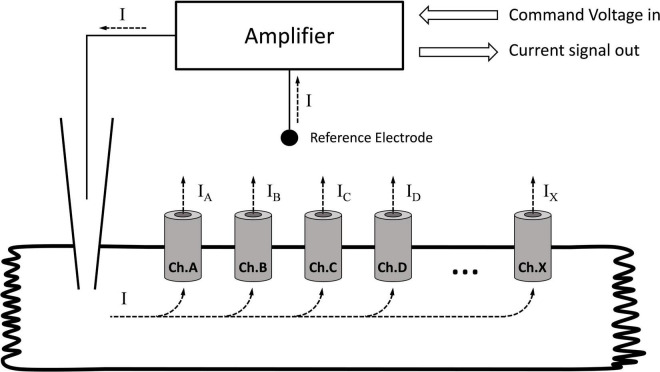
Recording the membrane current with the single electrode (discontinuous) voltage clamp technique. Ch.A, Ch.B, … Ch.X indicate different channel types in the membrane. The amplifier measures the total current passing through the membrane. If the membrane contains different channels, the current measured by the amplifier will be the sum of the individual currents. If the goal is to determine the current generated by a single channel type, all other channels must be turned off.

The voltage clamp technique was developed by Kenneth Cole and George Marmont in 1947 to study giant axon electrophysiology (Verkhratsky and Parpura, 2014). Silvio Weidmann combined this method with the glass microelectrode and adapted it to the cardiac myocyte (Kléber et al., 2006). Regardless of the size, or the type of the cell being voltage-clamped, the concept of this technique is simple. The glass microelectrode, filled with an electrolyte, establishes an electric contact between the inner space of the cell and the amplifier. The other pole of the amplifier is connected to the extracellular reference electrode immersed into the perfusion fluid. In this simple circuit, all elements are connected in a chain (i.e., series circuit); hence, the current measured with the amplifier will represent the current flowing through a membrane (Figure 1). If the membrane contains multiple ion channel types (i.e., sodium, potassium, and chloride), the total membrane current is a composite current. During a voltage clamp experiment, the amplifier maintains the voltage pattern (e.g., rectangular, ramp, and sine) determined by the command voltage and measures the current in the circuit that is the membrane current.

## Traditional Voltage Clamp Technique Using Rectangular Commands

The primary research goal when using rectangular command voltage is to characterize the behavior of ion channels and understand how the changing membrane voltage modulates their function. The function of ion channels is determined by the conformation of the pore-forming protein. The transition between functional states (i.e., short states) is governed by membrane potential changes. Using a series of rectangular command voltages (i.e., rapid voltage step followed by sustained voltage levels) the kinetic properties (i.e., activation, inactivation, and recovery) of a given channel can be mapped. Hypothetically, having all the channel parameters determined, the magnitude of the current can be predicted during any voltage change with an appropriate mathematical model (Xu et al., 2002; Bondarenko et al., 2004; Shannon et al., 2004; Romero et al., 2011). There is only one serious technical difficulty to be handled in these studies: dissecting the individual current from the total membrane current. Since the cardiac cell membrane contains numerous channels, before we can start recording the one single current we are interested in, all other channels must be “silenced.” Blocking all unwanted currents in cardiac cells is a difficult task. To achieve this goal, experimenters often use drugs or non-physiological conditions. The intracellular and extracellular ion species and concentrations are usually different from the physiological milieu during the traditional voltage clamp experiment. Then, the reconstruction of the current in the computer model is based on assumption that the channel behaves similarly in a physiological environment.

## Action Potential Voltage Clamp—Direct Measurement of Ion Current During Action Potential

**FIGURE 2:**
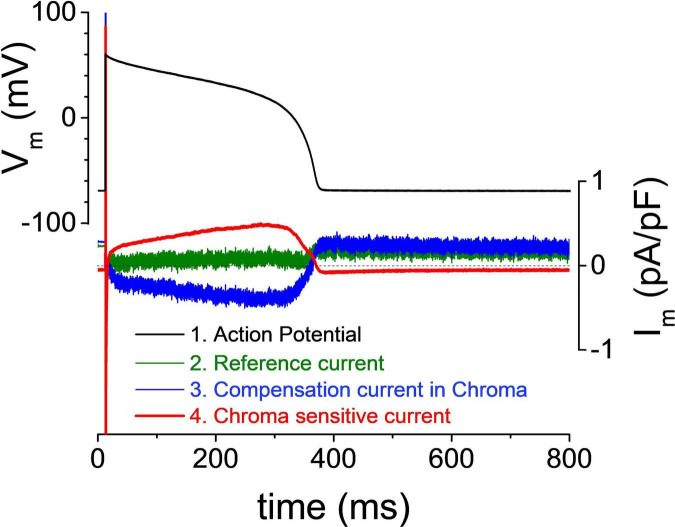
Self-action potential clamp recording of an ion current. The cell’s action potential (depicted in black) is used as command voltage. The reference current (depicted in green) is recorded immediately after steady state was achieved. After the application of Chroma (Chromanol-293B), I_*Ks*_ was blocked, and the compensation current (depicted in blue) was provided by the amplifier. The Chroma-sensitive current (depicted in red) was obtained by subtraction of the compensation current from the reference current.

The great quote “Measure what is measurable, and make measurable what is not so” attributed sometimes to Galileo Galilei, at other times to the French scholars Antoine-Augustin Cournot and Thomas-Henri Martin, could be a first-rate motto for this technique. This experimental method uses a “reverse approach” in order to determine the current profile directly during AP. To achieve this, a four-step protocol is employed. First, an AP is recorded from the cell in physiological ionic milieu under the current clamp mode. Second, the recorded AP is fed back to the amplifier as command voltage in the voltage clamp mode, and the compensation current is recorded. Under these circumstances, the compensation current will be a flat line with some minor artifacts before the upstroke caused by the exogenous stimuli in the previous step. In an ideal measurement, the compensation current will be zero because the cell membrane does not require any exogenous current from the amplifier to produce its own AP (Figure 2, green trace). This flat compensation current is saved and used later as reference current. In the third step, a selective ion channel blocker is applied to turn off the current to be recorded. If the compensation current is recorded following the full development of the inhibition, the mirror image of the blocked current will be seen. The current in interest is blocked, and the missing current is supplied by the amplifier (Figure 2, blue trace). In the fourth step, the second current trace is subtracted from the first. Since the first compensation current is practically a flat line in the optimal case, the subtraction results in flipping the second trace (Figure 2, red trace). Now, the produced trace reveals the current profile during the AP. Unlike in the first case, this trace is not a reconstruction but a direct record of the current studied in the physiological milieu.

## The Technical Evolution of the Concept

Action potential-shaped command voltages were already used for various purposes before the concept of the AP clamp was used for the current recording. Bastian and Nakajima employed prerecorded APs to study the T-tubule function in the skeletal muscle using the double sucrose gap method (Bastian and Nakajima, 1974; Nakajima and Bastian, 1974). Starzak et al. used the AP-shaped command pulse to test the effectiveness of the space clamp in the squid axon (Starzak and Starzak, 1978; Starzak and Needle, 1983). Although these authors made no attempt to record specific ion currents in these experiments, the essential elements of AP clamp technique can be identified. Remarkably, Starzak et al. consistently used the term “action potential clamp” in their publications and defined an essential criterion for the technique: “To produce the most accurate reproduction of this AP, the voltage clamp currents must include no contributions due to ineffective space clamp” (Starzak and Starzak, 1978).

Attempts to record ion currents directly during the AP can be traced back to the early 1970s. Bezanilla et al. (1970) published the first method in 1970 to record membrane conductance during AP in the squid axon. The cell was stimulated by a brief current pulse, and then, the AP was allowed to develop in the current clamp mode. At the required point, the amplifier was switched to the voltage clamp mode in less than 10 μs and the current-voltage relationship of the membrane was determined. The instantaneous current-voltage relationship made it possible to obtain a good estimate of the sodium and potassium conductance at different points of the AP. The technique was adapted and improved by several followers, and at present, it is termed a discontinuous single-electrode voltage clamp (dSEVC) (Brennecke and Lindemann, 1971, 1974; Wilson and Goldner, 1975; Merickel, 1980).

Another approach employed two recording electrodes but not prerecorded AP (Jackson et al., 1982; Fischmeister et al., 1984; Mazzanti and DeFelice, 1987). The first electrode penetrated the membrane and accessed the interior of the cell; this electrode recorded the membrane potential in the current clamp mode. The other electrode was sealed to the membrane surface and recorded the current passing through the membrane patch under the tip in the voltage clamp mode. The high access resistance prevented clamping the whole cell. The cell can be stimulated *via* the voltage recording electrode, and the cell produces the AP in the “free running mode.” The measured voltage served as the input voltage value for the second electrode. The method has two important limitations. First, the patch under the current recording electrode has usually a few ion channels. Second, the natural instability of the AP parameters increases the variability of the recorded current.

A big breakthrough came in 1989 with two pioneering publications from two independent teams. Doerr et al. used digitized AP in spontaneously beating rabbit sinoatrial node cells as command voltage and recorded the compensation current (Doerr et al., 1989). Similar experiments were conducted by de Haas and Vogel (1989) in single nerve fibers of *Xenopus laevis*. Specific channel blockers and current subtraction were used to dissect the current of interest during AP in both works. These publications reported the first current profiles obtained with direct recording during AP. The method gained popularity in a short time and grew into a valuable tool for mapping the ion currents that shape AP in various cells types including the cardiac myocytes (Doerr et al., 1990; Bouchard et al., 1995), neurons (Barra, 1996), and plant cells (Thiel, 2004). The subtraction method is used with various command pulses including rectangular steps or ramps especially to isolate small currents (Wagner et al., 2006; Maltsev and Kyle, 2009; Ton et al., 2021). Combined with fluorescent techniques, the AP clamp method became a powerful tool for studying the Ca^2+^ dynamics in cardiac myocytes (Arreola et al., 1991; Grantham and Cannell, 1996; Puglisi et al., 1999).

For a long time, the AP clamp was used to record only one single current from one cell. In this respect, it did not differ from the traditional voltage clamp method. Limitation resulted from the fact that in AP clamp experiments, the stability of the AP is judged by the flatness of the reference current after the seal is established. If the compensation current is not a flat, horizontal line, this is the indication that no steady state is reached, and the cell is not stable. Deviation of the current from the zero level to either positive or negative direction (“bumps” or “pits”) results from changing the current magnitude during the AP. For a long time, nobody attempted to apply a second or third channel inhibitor upon the cell after the development of the effect of the first drug. The first report using multiple channel inhibitors in a sequential manner to record multiple currents in the same cell was published in 2011. The new method, named as “sequential dissection technique,” added only one innovative idea to the regular AP clamp method: multiple channel inhibitors were applied cumulatively, and the compensation current, recorded after the development of the effect of a channel inhibitor, was used as a reference current for the next compensation current (Banyasz et al., 2011). Hypothetically, this new advancement allowed the experimenter to determine all current during the AP, assuming that each current has a specific inhibitor.

## Variants of the Action Potential Clamp Technique

Several variations of the AP clamp technique are used in various labs these days. These variants employ different modifications in order to circumvent technical limitations or extend certain capacities of the method. Discussing all possible modifications within one single article would be an overambitious aim leading to the loss of the focus of our article; therefore, we restricted ourselves to outline the main variants only.

## Using Self-Action Potential, Canonical Action Potential, or Modified Action Potential as Command

Individual cells display distinctive APs with some degree of cell-to-cell variations. The selection of the AP influences the current profiles obtained in the experiments. Using the cell’s own AP is an obvious choice resulting in a decent, flat reference current in the first phase of experiments. This method is referred to as the self-AP clamp technique (^Self^AP clamp) (Hegyi et al., 2020). The essence of this technique is to maintain the cell’s own steady-state AP by satisfying the following requirements. First, the physiological ionic composition is used in both internal and external solutions containing no exogenous calcium buffer. Second, the cell is paced sufficiently long at a constant rate to stabilize AP prior to the current recording. These two conditions maintain the natural flow of ion currents and calcium cycling. Therefore, the recorded currents reflect the natural profile of the currents during the AP of the actual cell patched. The leading disadvantage of the self-AP clamp originates from the cell-to-cell variation. The AP parameters (e.g., length and plateau height) show considerable cell-to-cell variation that translates to increased variability of the currents due to the high-voltage sensitivity of the activation and inactivation of the ion channels. For instance, a few millivolt elevation of the plateau height was reported to increase potassium currents substantially in canine ventricular myocytes (Horvath et al., 2006). Another disadvantage results from using pipette solution that mimics the physiological intracellular ionic milieu. Traditionally, ethylene glycol-*bis*(β-aminoethyl ether)-*N*,*N*,*N*’,*N*’-tetraacetic acid (EGTA) is added to the internal solution to buffer calcium in voltage clamp experiments. Buffering cytosolic calcium eliminates the contraction of the cell, increasing the lifetime of the seal. Using “physiological” internal solutions without calcium buffer leave the cell’s calcium cycling intact, and the cell will develop regular contractions during the experiment.

Using canonical (i.e., “typical,” “average,” and “standardized”) AP can prevent the error introduced by the cell-to-cell fluctuation and offers reduced variability for the recorded current (Hezso et al., 2021; Horváth et al., 2021). An undesirable consequence of using canonical AP is that the reference current is not a flat line. Thus, it is challenging to judge the stability of the AP after the seal is established. Rundown of currents or other types of electric instabilities of the cell causes growing deflection or hump on the reference current. These changes are easier to recognize when the reference current is a straight line, like in the case of the self-AP clamp.

Modified APs can be used for various reasons in AP clamp experiments. One possible reason for using modified AP is to circumvent technical difficulties, like possible conflict between the resolution of the recording and voltage clamp fidelity. When small currents (<1 nA), like I_Ks_ or I_Kr_, are recorded in the cardiac cells, it is practical to set the amplifier gain high in order to increase the resolution and record fine details of the current profile. Unfortunately, increasing the gain reduces the maximum output current of the amplifier. Therefore, high gain increases the chance to lose voltage control during the upstroke of the AP where sodium current may exceed 100 nA. The consequences are serious because the membrane voltage is uncertain until the control is not regained. There are two equally poor options here. If the amplifier gain is kept high, the resolution is maintained, but the clamp would be lost at the beginning of the AP. If the gain is set low, the voltage control is maintained, but the resolution of the recorded current is poor. The solution could make use of modified AP with a short, depolarizing voltage step (10 ms, −30 mV) prior to the upstroke. This depolarizing step can inactivate the voltage-activated sodium channels allowing the amplifier to maintain voltage control during the whole AP (Varro et al., 2000). Custom-tailored APs with modified length, plateau height, or diastolic interval can also be used to study the dynamics of ion currents that shape AP (Rocchetti et al., 2001).

## Dynamic Clamp

A unique variant of the AP clamp method is the dynamic clamp, where the AP command for the voltage-clamped cell (Cell 1) is obtained from a current clamped cell (Cell 2) or mathematical model (Berecki et al., 2005; Meijer van Putten et al., 2015). Then, the current recorded in Cell 1 is fed back to Cell 2 (or the mathematical model), so it can modify the morphology of the original AP. In this configuration, the two systems (cells) are in dynamic connection and real-time coupling. The dynamic clamp technique provides a powerful tool for studying the dynamic interaction between a current and the AP. For example, Mahajan et al. used the dynamic clamp method to investigate the Ca^2+^ modulation of ionic currents during AP in the cardiac myocyte (Mahajan et al., 2008). First, they eliminated Ca^2+^ cycling by depleting the sarcoplasmic reticulum, and the calcium transient was supplied by a mathematical model. The data were fed into the AP-clamped cell, and they recorded the L-type calcium current. Using this technique, they could explore the interaction between the calcium transient and L-type calcium current during AP. The greatest advantage of the dynamic clamp method is that the AP obtained from the current-clamped cell or mathematical model can be manipulated, and the interaction between the currents and the voltage profile can be studied. Furthermore, it can be determined how different ion currents communicate *via* the voltage during AP.

## Sequential Dissection Action Potential Clamp or “Onion-Peeling” Technique

**FIGURE 3:**
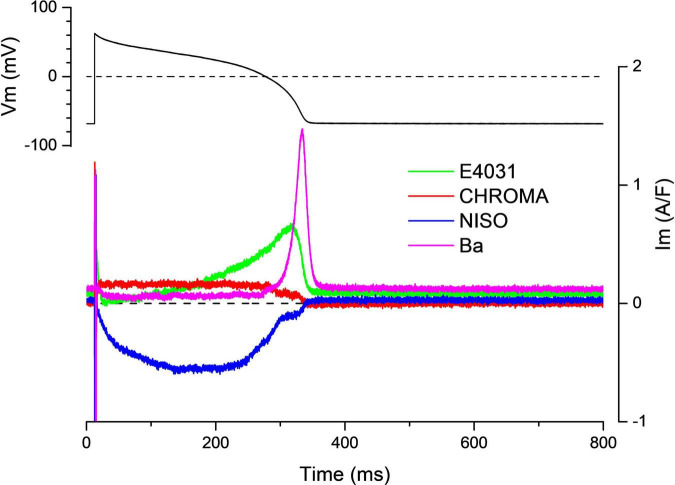
Onion-peeling recording of multiple currents in the guinea pig ventricular myocyte. Representative current set.

This further developed version of the self-AP technique allows the recording of multiple ion currents from the same cell (Banyasz et al., 2011, 2014; Horvath et al., 2013). Before the current recordings, the cell’s AP is recorded under the current clamp condition. This AP is used during the whole experiment as the command voltage. Then, the reference current is recorded, and the first channel blocker is applied. Following the development of the drug effect, the compensation current is recorded. In a regular self-AP clamp measurement, the experiment is finished and the cell is discarded. Using the onion-peeling technique, the experiment is continued with the next channel blocker added upon the first blocker. After the development of the effect, the compensation current is recorded again. The cumulative application of the channel blockers can be continued *ad libitum*. During the data processing, any current can be determined by the subtraction of the compensation current recorded under the effect of its blocker from the previously recorded compensation current. Hypothetically (if the viability of the cell is appropriate), it is possible to record each current of the membrane during the AP. Figure 3 shows four current traces recorded in a guinea pig cell during the AP with this method. Conventionally, these currents are referred to as drug-sensitive currents in the literature. Hence, the current dissected out with E4031 is termed “E4031-sensitive current” not I_Kr_. The reason for this distinction is that the selectivity of pharmacological inhibitors is sometimes debated. Table 1 lists channel inhibitors known to have sufficiently high selectivity.

**TABLE 1:** Specific inhibitors for the major ionic currents in cardiac cells.

Ion Channel and transporter	Inhibitor	Effective concentration	References
I_Na_	TTX	1–10 μM	Yuill et al., 2000; Chorvatova et al., 2004
I_Na–Late_	Ranolazine	10 μM	Rajamani et al., 2009
I_Na–Late_	Eleclazine (GS-6615)	10 μM	Rajamani et al., 2016
I_Na–Late_	GS-967	1 μM	Potet et al., 2020
I_Ca–L_	Nifedipine	1 μM	Horiba et al., 2008
I_Ca–L_	Nisoldipine	0.1 μM	Banyasz et al., 2003
I_Ca–T_	NNC 55-0396		Huang et al., 2004
I_Ca–T_	R(-)efodipine		Tanaka et al., 2004
I_NCX_	SEA0400	3 μM	Birinyi et al., 2005; Ozdemir et al., 2008
I_to_	4-aminopyridine	1 mM	Patel and Campbell, 2005; Banyasz et al., 2007
I_kr_	E4031	1 μM	Varro et al., 2000; Banyasz et al., 2007
I_ks_	HMR-1556	30 nM	Gögelein et al., 2000; Thomas et al., 2003
I_ks_	Chromanol-293B	1–10 μM	Yamada et al., 2008
I_k1_	BaCl_2_	50 μM	Banyasz et al., 2007; Banyasz et al., 2008
I_K–Ca_	Apamin	100 pM, 1 nM	Xu et al., 2003; Ozgen et al., 2007
I_Cl–Ca_	Niflumic acid	50 μM	Greenwood and Leblanc, 2007; Saleh et al., 2007
I_Cl–Ca_	N-(p-amylcinnamoyl) anthranilic acid	5 μM	Gwanyanya et al., 2010
I_Cl–small_	Chlorotoxin		Borg et al., 2007
I_Cl–vol_	Tamoxifen		Borg et al., 2007
I_Cl,ligand_	Picrotoxin		Etter et al., 1999

The quality requirements are exceptionally high to successfully perform onion-peeling experiments. Robust cells with stable membrane potential and AP are vital. Cell isolation is basically the same as in other single cell methods, but the storage media should mimic the physiological milieu. If any media with non-physiological ionic composition are used during the cell isolation, the exposition time should be minimized. The pH and osmolarity of the solution must be tightly controlled, and using membrane-permeable buffers is known to increase the robustness of the cells. The pipette solution used in these experiments should mimic the intracellular ionic milieu of the cell. Pipette resistance and noise should be kept low. Since the pipette solution contains no exogenous calcium buffer, each AP evokes contraction of the cell; hence, the seal must be sufficiently stable to survive hundreds of contractions during the experiment. The instrumentation (i.e., A/D converter and stimulator) used in these experiments are basically the same as for any voltage clamp setup used for recording small (<1 nA) currents. A good external square wave/pulse generator is very useful in these experiments. Although most electrophysiology software offers the option of stimulating the cell with a rectangular pulse in current-clamp mode, adjustment of stimulation parameters is complicated. Old-fashioned external square pulse generators usually have control knobs that allow the experimenter to adjust any parameters without interrupting the actual experimental protocol.

## The Potential of Action Potential Clamp Technique in Integrative Physiology

Cardiac arrhythmias are the primary cause of death in the developed world. Pathological conditions that serve as a substrate for arrhythmias are consequences of complex changes in ion channels/transporters and calcium handling molecule function. The dysfunction of these systems leads to alteration of one or more membrane currents causing abnormal AP generation with resultant arrhythmias. To understand how altered membrane currents with the deformed AP cause pathological rhythm generation, one needs to understand the interaction of the individual ion currents during the AP. The traditional approach to achieve this goal was combining voltage clamp experiments with mathematical modeling. Choosing this method inherently assumes that it is possible to determine the sufficient number of parameters with the required accuracy with rectangular command voltages for each ion current to successfully reconstruct the AP with computer model. Since the parameters of the individual currents are obtained in different experiments, *post hoc* adjustment/optimization is often needed during the model construction. The AP clamp technique determines the current profile directly during the AP without later adjustment of the obtained data.

## Pharmacological Application: Identifying the Target(s) of Unknown Drugs

Each ionic current has a characteristic profile/trajectory during AP termed as fingerprint. The AP clamp technique is suitable for economic screening of new drugs with unknown target(s). After having all ion currents mapped, the suspected drug-sensitive current can be identified in a single AP clamp experiment (Szabo et al., 2008; Szentandrassy et al., 2011). Analyzing the profile of the drug-sensitive current recorded with the AP clamp can give a clue toward the identity of currents that might be affected by the drug. Prescreening a drug with an unknown effect prior to systematic pharmacological tests can save valuable time. Effects of receptor agonists/antagonist or hormones can also be readily screened with the AP clamp. Even in the case when an agonist/antagonist affects only one population of receptors, signal transduction pathways often couple to more than one ionic current; the AP clamp is uniquely suited for studying the drug concentration-dependent effects on multiple currents. As example, the frequency-dependent effects of isoproterenol on I_Ca,L_, I_Ks_, and I_Cl_ in guinea pig ventricular cells (Rocchetti et al., 2006) and the effects of acetylcholine and adenosine on I_K1_ in ferret cardiac myocytes (Dobrzynski et al., 2002) were clarified using the AP clamp technique.

## Regional Differences Within the Heart/Study of the Individual Cell Electrophysiology

Action potential morphology has long been known to have characteristic regional differences within the heart. Apico-basal and transmural differences in AP morphology were demonstrated in several species (Banyasz et al., 2003; Szentadrassy et al., 2005). This inhomogeneity results from varying current densities in the different regions. When L-type calcium currents I_Ca,L_ of epicardial and endocardial cells were compared with the traditional (i.e., rectangular pulse) voltage clamp technique, the voltage dependence, activation, and inactivation kinetics of the currents were found not statistically different. Interestingly, when the profile of I_Ca,L_ was determined as nisoldipine-sensitive current with the AP clamp technique, marked differences were observed between epicardial and endocardial cells (Banyasz et al., 2003). I_Ca,L_ exhibited a sharp spike followed by a rapid decay in both endocardial and epicardial canine ventricular cells following the upstroke of the AP. Following the spike, a hump developed on the I_Ca,L_ was recorded in epicardial but not in endocardial cells. This hump, or second peak, arose following the deepest point of the incisura and reached its maximum during the crest of the dome. The amplitude of the first peak was greater in endocardial than in epicardial cells, and the amplitude of the second peak was smaller than the first one. No sustained current was recorded during the plateau in endocardial cells, in contrast to the slowly declining but non-zero current flowing during the dome of epicardial myocytes. There was a tight correlation between the parameters of AP and I_Ca,L_, indicating that the development of the AP dome and the second I_Ca,L_ peak are coupled tightly. This suggests that the second peak on I_Ca,L_ may provide the depolarizing current responsible for the formation of the dome under physiological and development of EADs under pathological circumstances. The second activation of I_Ca,L_ is also clearly demonstrated by the current-voltage relationship, obtained under AP clamp conditions. Essentially, similar results were obtained in ventricular myocytes dispersed from healthy human hearts (Fulop et al., 2004). This double-peaked performance of I_Ca,L_ in epicardial canine and human ventricular cells and the relationship between the profile of AP and I_Ca,L_ can be demonstrated exclusively under AP clamp conditions but not with the traditional voltage clamp method. Furthermore, these data established the first time direct connection between the trajectory of a membrane current and the AP.

Regional differences are not the only electrophysiological inhomogeneity within the myocardium. It has been widely known that cell-to-cell variations exist within small regions of the myocardium. Traditional voltage clamp studies investigate one current from any one cell and different current from different cell. Then, averaged data from multiple cells are used to construct canonical AP models. Nonetheless, the averaged canonical AP model may not reflect the behavior of all cells due to cell-to-cell variability (Marder and Taylor, 2011; Pathmanathan et al., 2019). It has been evident that discrepancies exist between model simulations, and the AP clamp measured current and model simulations still fail to reproduce some of the AP dynamics including EAD, adaptation, and restitution (Faber et al., 2007; Mahajan et al., 2008; Pasek et al., 2008; Decker et al., 2009; Banyasz et al., 2011). It is the unique potential of the onion-peeling technique that it can record the currents during individual AP providing input data accurate and realistic models allowing to study the interaction of currents in individual cells.

The onion-peeling technique allows to correlate AP parameters with membrane currents. In an earlier study, our team studied the membrane currents during the AP in guinea pig ventricular myocytes (Banyasz et al., 2011). Four currents, namely, nisoldipine-sensitive current, Chromanol-293B-sensitive current, E4031-sensitive current, and Ba^2+^-sensitive current, were recorded. Cardinal parameters of the current traces (i.e., peak amplitude, time to peak, and charge movement) were analyzed and compared. Analysis results showed that some cells had large current density for all four currents recorded, while others had small ones. The values span a wide range; for example, the ratio of the largest and smallest nisoldipine-sensitive current was higher than 10. The difference could not result from the cell size because all current magnitudes were normalized to cell capacitance. Data showed that cells exhibiting large inward currents also had large potassium currents. The statistical analysis revealed linear correlation between the inward going charge carried by Ca^2+^ and the outward moving charge carried by the sum of potassium currents during the AP. Interestingly, the AP parameters (i.e., length and plateau height) showed no correlation with the current magnitudes or the moving charge. These data provide the first observations to demonstrate that either large or small currents can generate similar APs as long as the inward going and outward going currents counterbalance each other in the cell. The possibility to measure multiple currents from the same cell has revealed hitherto unknown and unexpected characteristics of the ionic currents in myocardial cells. First, within a single cell, large outward current/charge movement is matched with large inward current/charge movement and vice versa; hence, the magnitudes of the depolarizing and repolarizing currents seem to be coordinated in such a way as to produce canonical AP, as seen in healthy guinea pig ventricular myocytes in this study. Second, there exists large variation in the current/charge movements between cells. These two findings suggest a more pluralistic view of cardiac cells. The magnitude of ionic currents can vary widely between cells, but the relative strength of the depolarizing and repolarizing currents is somehow constrained within a cell to produce similar APs. Variation and coordination of ionic currents in cardiac myocytes should have a significant impact on the AP and play important roles in arrhythmias.

## Coordination of Membrane Currents

Cardiac membrane currents are differentially modulated by various control mechanisms including sympathetic stimulation or humoral factors. Thus, the relative contribution of the individual currents to the AP may change during cardiac adaptation. The onion-peeling technique is a feasible method to study the changing balance among membrane currents in various conditions. For example, there is a consensus on that I_Kr_ and I_K1_ are the two chief repolarizing currents in the ventricular myocyte; the contribution of I_Ks_ to repolarization is minor. β-adrenergic stimulation is known to facilitate these currents in different extent. I_Ks_ is reported to be very sensitive to β-adrenergic stimulation; the sensitivity of the other two currents is less. In all previous studies, however, each potassium current was recorded from different cells and using different voltage clamp protocols. Thus, it remains unknown how β-adrenergic stimulation coordinately regulates these current in the same cell during AP. Previous experiments could not answer the question how β-stimulation changes the relative contribution of each potassium current to the repolarization reserve. Such knowledge is essential for designing antiarrhythmic strategies involving potassium channel blockers. Using the onion-peeling method, it became possible to measure and analyze the proportion of different potassium currents in the same cell during the AP in control condition and following β-stimulation (Banyasz et al., 2014). According to the data obtained, isoproterenol greatly increased I_Ks_, making it to the most powerful repolarizing current in guinea pig cardiac myocytes. I_Kr_ was insensitive to isoproterenol, so its contribution to the repolarizing reserve was reduced. At high isoproterenol concentrations, the contribution of I_Ks_ surpassed the I_Kr_ contribution by 4- to 5-folds. The I_K1_ magnitude was marginally increased, but its relative contribution was unaltered. Previously, the contribution of different potassium current to the cell’s repolarization power has been a subject of debate. The onion-peeling method can provide accurate measure to characterize the coordination of the three potassium current during the AP.

The onion-peeling technique was used to explore the coordination between inward and outward currents in a recently published work by Hegyi et al. (2020). Four inward currents and six outward currents were recorded and analyzed in rabbit and porcine ventricular cells. The authors demonstrated that the I_Kr_ and the late sodium current (I_Na,L_) counterbalance each other during the ventricular AP. That is, robust linear correlation was found between the peak amplitude and the total moving charge of these two currents under various conditions. The correlation was present in young and old control animals and in a heart failure model as well. The correlation between these two currents was demonstrated after prolongation of the AP with anemone toxin II too. Additionally, the authors reported a strong correlation between the length of the AP and the current parameters (i.e., peak density and total charge movement). These correlations should be considered when building *in silico* AP models. Recently, Pathmanathan et al. (2019) demonstrated that correctly accounting for these types of correlations may be the key to successful uncertainty quantification when evaluating computational models.

## Concluding Remarks

This review summarizes the development of the voltage clamp technique from the traditional version that applies rectangular command to the onion-peeling method. These new techniques widen the opportunities for exploring the function of individual ion channels as well as the coordination of membrane currents during AP. The onion-peeling technique is exceptionally suited for studying the interplay among the currents; thus, it has a unique potential in integrative physiology. It was developed for cardiac myocytes but can be adapted to the skeletal muscle or smooth muscle cells, neurons, and any excitable cell type where ionic currents and AP control cell function.

## Author Contributions

BH, NS, CD, ZK, PN, YC-I, and LI contributed to conception and design of the work, literature search, and review of the text. TB contributed to conception and design of the work, literature search, drafting, writing, and figure design and drawing. All authors contributed to the article and approved the submitted version.

## Conflict of Interest

The authors declare that the research was conducted in the absence of any commercial or financial relationships that could be construed as a potential conflict of interest.

## Publisher’s Note

All claims expressed in this article are solely those of the authors and do not necessarily represent those of their affiliated organizations, or those of the publisher, the editors and the reviewers. Any product that may be evaluated in this article, or claim that may be made by its manufacturer, is not guaranteed or endorsed by the publisher.

## Abbreviations

AP: action potential
I_Ca,L_: L-type calcium current
I_K1_: inward rectifying potassium current
I_Kr_: rapid component of delayed rectifier potassium current
I_Ks_: slow component of delayed rectifier potassium current
I_Na,L_: late sodium current.
